# The Effects of Social Media Addiction, Psychological Distress, and Loneliness on Suicide Ideations and Attempts Among Healthcare Professionals in Saudi Arabia

**DOI:** 10.7759/cureus.44234

**Published:** 2023-08-28

**Authors:** Mahmoud A Mahmoud, Khalid T Abolashamat, Baraa S Quronfulah, Mona T Rajeh, Amal M Badawoud, Abdullah M Alzhrani, Ismail M Abdouh, Hatim M Badri

**Affiliations:** 1 Public Health, Imam Muhammad Ibn Saud Islamic University, Riyadh, SAU; 2 Preventive Dentistry, Umm Al-Qura University, Makkah, SAU; 3 Health Promotion and Health Education, Umm Al-Qura University, Makkah, SAU; 4 Public Health Dentistry, King Abdulaziz University, Jeddah, SAU; 5 Pharmacy Practice, Princess Nourah Bint Abdul Rahman University, Riyadh, SAU; 6 Occupational Health, Umm Al-Qura University, Makkah, SAU; 7 Oral Basic and Clinical Sciences, Taibah University, Al Madinah Al Munawara, SAU; 8 Environmental Health, Umm Al-Qura University, Makkah, SAU

**Keywords:** depression, loneliness, suicide attempt, suicide ideas, psychological distress

## Abstract

Introduction: Recently, there has been an alarming increase in psychological distress in many populations. One of the reasons can be attributed to the rapid development of technology and social media, which could adversely affect the mental health of individuals, including those working in healthcare. This study aimed to assess the influence of social media addiction, psychological distress, and loneliness on suicidal ideations and suicide attempts among healthcare students and professionals in Saudi Arabia.

Materials and methods: This cross-sectional study collected data from healthcare students and professionals using a five-part questionnaire: (i) demographics, (ii) the Bergen Social Media Addiction Scale (BSMAS), (iii) the Kessler Psychological Distress Scale (K10), (iv) the De Jong Gierveld Loneliness Scale (DGLS), and (v) suicide ideation and attempts scale.

Results: There were 800 participants from 33 cities who completed the questionnaire. A total of 31.37% reported lifelong thoughts of suicide, regardless of whether they would actually go through with it (S1), 18.38% had suicidal thoughts within the last 12 months (S2), and 11% had attempted suicide (S3). Of those who attempted suicide, 79 (89.77%) reported seeking help. Multiple logistic regression showed that lifetime suicidal thoughts were predicted by psychological distress, emotional loneliness (EL), social loneliness (SL), and age. Having had suicidal thoughts within the prior 12 months was predicted by psychological distress, SL, and age. Suicidal attempts were predicted by psychological distress, EL, age, and social media addiction.

Conclusions: Our findings demonstrated that psychological distress and loneliness are strongly associated with suicidal ideas and suicide attempts. Such results could serve as a warning call that assists healthcare professionals and mental health teams in arranging and planning effective interventions and actions to raise awareness, as well as reduce the levels of psychological distress and loneliness that could lead to grave consequences.

## Introduction

Social media platforms have transformed the way people connect; through advancements in technology, people connect with whomever they want wherever they are. Platforms such as Twitter, WhatsApp, and Instagram have become the predominant shape of communication worldwide [1]. Although social media are useful for networking, information retrieval, and dissemination of knowledge to patients, they were found to be addictive and adversely affect psychological and social well-being [2]. It has previously been reported that over 90% of healthcare professionals utilize social media [3], over 75% of healthcare workers use social media at work [4], and more than 40% of healthcare buyers rely on social media for their data needs [3].

Some articles suggest that social media addiction may increase an individual’s risk of anxiety and depression [2]. Since its inception, social media has seen increasing addiction rates. Hence, depression, anxiety, and loneliness rates have increased as well [5]. In one study of 2,000 healthcare practitioners, around 27.3% suffered severe psychological stress [6]. According to a recent meta-analysis of 35 studies and 70,368 physicians, the lifetime prevalence of suicide attempts was 1.8%, while the one-year prevalence was 0.3% [7].

Studies examining suicide thoughts among medical students worldwide have revealed varying findings; however, rates have been reported that cause concern and demand attention and intervention on a broad scale. Using information from 24 studies in 15 countries, a meta-analysis reported that the crude prevalence of depression or depressive symptoms was 27.2%, the percentage of medical students screening positive for depression who sought psychiatric treatment was 15.7%, and the overall pooled crude prevalence of suicidal ideation was 11.1% [8].

In a study from the United States, suicidal ideation among medical students ranged from 1.4% to 10.3% [9]. A study from the United Arab Emirates reported similar rates, with suicidal ideation ranging from 1.8% to 17.5% [10]. A study from Taiwan, however, found significantly higher rates of 43.6% [11]. Although there have been some reports about suicide ideation in Saudi Arabia, the precise prevalence is yet unknown. A small descriptive study aimed at assessing the prevalence of suicidal thoughts among medical students in the Eastern Province of Saudi Arabia reported that 42.2% had suicidal ideation in their lifetime, and 35.4% reported thoughts of suicide within the last 12 months [12]. Another study conducted in Abha found that among dental students, 43.5% had suicidal ideation in their lifetime, 30.7% reported it in the last 12 months, and 15.1% reported attempting suicide [13].

Most studies that focused on the effects of social media addiction on suicidal ideation were conducted with the general population [14]. To our knowledge, no investigations have yet been conducted regarding the impact of social media addiction on suicidal ideation among healthcare students and professionals. Thus, the present study aimed to explore the effects of social media addiction, psychological distress, and loneliness on suicidal ideations and suicide attempts among healthcare students and professionals in Saudi Arabia.

## Materials and methods

Study design and participants

As part of a national research project, this study aimed to assess the psychological health and lifestyle practices of healthcare students and professionals. The process for conducting this study was similar to the processes used in other studies being conducted in connection with the main project. The study's sample size was determined based on a CI of 95%, an estimated prevalence of 50%, and a desired margin of error of 5%. Using these parameters, the calculated minimum sample size was 386 participants.

For this cross-sectional study, an online questionnaire was distributed throughout the Kingdom of Saudi Arabia. The inclusion criteria for the participants included healthcare professional workers, healthcare students, graduates, and interns from the Colleges of Pharmacy, Applied Medical Sciences, Dentistry, Nursing, and Medicine, which were the data sources for this study. They must be living in Saudi Arabia. The exclusion criteria included those who did not approve the consent form and those who retired. The survey was open for three months, from April 6, 2022, to June 1, 2022. Convenience sampling was used to gather the data. Participants were recruited and given the questionnaire via personal invitations sent through social media platforms, such as Twitter, Snapchat, WhatsApp, and other applications, along with a cover letter consent form.

Participation was voluntary, with no incentives, and participants had the option to withdraw at any time. All responses were anonymous, with no tracking of email addresses or any other identifying information. The data for this survey were stored on private devices that are access-restricted and available only to the survey team.

Study measures

The questionnaire consisted of 32 questions spanning five sections to measure demographics, psychological distress, loneliness, social media addiction, suicidal ideation, and attempted suicide. The first section gathered demographic information, including nationality, health discipline, age, gender, qualifications, region, city, chronic illnesses, and healthy eating.

In the second section, we assessed psychological distress levels using the Kessler Psychological Distress Scale (K10) [15,16], which comprises 10 items answered on a Likert scale from 1 (not always) to 5 (always). The total scale score ranged from 5 (minimal distress) to 50 (maximum psychological distress) [17]. K10 is an efficient tool for screening psychometric properties and has excellent internal consistency (validated in Arabic; Cronbach’s alpha = 0.88) [18,19].

Social media addiction was assessed in the third section via the Bergen Social Media Addiction Scale (BSMAS). This scale was originally designed to measure a leading social media platform [20]. Respondents were asked to rank their level of agreement with six statements using a 5-point Likert scale from 1 (very rare) to 5 (very often). Each participant’s scores were totaled to yield a new variable, the BSMAS total score, which ranged from 5 (no addiction at all) to 30 (highest level of social media addiction).

The fourth section assessed loneliness using the De Jong Gierveld Loneliness Scale (DGLS) [21], which encompasses two subscales of three questions each measuring emotional loneliness (EL) and social loneliness (SL). The DGLS score is the total of the scores on the two subscales. Each question is answered with one of three options: yes, more or less, or no. The coding into numbers for EL is different than for SL, as detailed in the questionnaire manual [21]. The subscale scores can vary from 3 (most lonely) to 0 (least lonely). The loneliness scale has a Cronbach’s alpha of 0.69 to 0.76 [21].

In the fifth, and final, section, we assessed suicide ideation and attempts through four questions, three of which were adopted from a previous study [22]. These questions, answered with yes or no, were as follows: “Have you ever had thoughts of taking your own life, even if you would not really do it?", “During the past 12 months, have you had thoughts of taking your own life?”, “Have you ever attempted to take your own life?”, and “If you have thought about taking your life or attempted that, did you ask for help?”.

This study adhered to the Declaration of Helsinki. Prior to the study, ethical approval was obtained from the ethical research committee of the Institutional Review Board (IRB) of the University of Umm Al-Qura, Saudi Arabia, which approved this project (HAPO-02-K-012-2022, Project No. 04-1048). The BSMAS, EL, SL, and suicide ideation and attempts sections of the questionnaire were translated from English into Arabic. This was followed by validation in a pilot testing phase. The pilot involved 13 participants who had the same eligibility criteria as this study and tested the questionnaire in terms of understanding, syntax, language, grammar, and organization. Based on the pilot study analysis, the questionnaire was modified according to our research objectives and revised to overcome all obstacles faced in the pilot study.

Statistical analysis

We analyzed the data using Microsoft Excel (Microsoft Corp., Redmond, WA, USA) for data entry and cleaning, in addition to SPSS Statistics version 27 (IBM Corp. Released 2020. IBM SPSS Statistics for Windows, Version 27.0. Armonk, NY: IBM Corp) for the statistical part. Descriptive statistics (i.e., frequencies, percentages, means, and SDs) were employed to summarize, describe, and prepare the analysis of the data. The chi-square test, Fisher’s exact test, and logistic regression were used to conduct the inferential statistical analysis. The three suicide questions were analyzed using multiple logistic regression to produce predictive models. The variables included in each initial model were gender, nationality, specialty, qualifications, the practice of eating healthy food, having a chronic disease, age, psychological distress (K10 score), social media addiction, EL, and SL. Then, backward elimination methods were used to produce the final model. We used a p-value <0.05 and a 95% CI to report the statistical significance and estimates in this study.

## Results

Demographic characteristics of participants

The data were collected and analyzed from 800 participants who responded appropriately to all sections of the structured questionnaire. The participants were distributed across 33 cities in Saudi Arabia, including Makkah, Riyadh, Jeddah, Madinah, Baha, Jazan, Abha, Dammam, Taif, Khobar, Qatif, Buraidah, Najran, Umluj, Buljurashi, Dahran, Khames Mushait, Kharj, Qassim, Skaka, Tabok, Yanbu, Ahsa, Badaea, Badr, Beshah, Hafr Albaten, Muhayel, Ras Tanura, Shaqra, Traif, Unyzah, and Wajh. The mean age of participants was 25.85 years (±8.25 SD). The highest percentage of respondents came from the Western region (61.5%) and the Central region (25.5%), followed by the Southern (9%) and Eastern (2.75%) regions and, finally, the Northern region (1.25%). There were more female respondents (59.88%) than males (40.13%). A majority of the respondents were students (56.63%), followed by interns/residents (24.38%) and specialists or consultants (19%). A majority of the participants had no chronic disease (86.38%) (Table 1).

**Table 1:** Demographic characteristics of the study sample

		Count	%
Gender	Male	321	40.13
	Female	479	59.88
Nationality	Saudi	771	96.38
	Non-Saudi	29	3.62
Region	Western	492	61.50
	Central	204	25.50
	Southern	72	9.00
	Eastern	22	2.75
	Northern	10	1.25
Specialty	Dentistry	181	22.63
	Public health	268	33.50
	Pharmacy	105	13.13
	Medicine	161	20.13
	Applied medical science	58	7.25
	Nursing	27	3.38
Qualification	Student	453	56.63
	Intern/resident	195	24.38
	Specialist or consultant	152	19.00
Eat healthy food	Yes	331	41.38
	No	469	58.63
Have a chronic disease	Yes	109	13.63
	No	691	86.38

Levels of suicidal thoughts and attempted suicides

Table 2 provides the data on suicidal thoughts and suicide attempts by demographics, psychological distress, loneliness, and social media addictions. About 31.37% of participants reported having ever thought of taking their lives, even if they would not follow through (S1), while 147 (18.38%) had thought of taking their own lives during the past 12 months (S2), and 88 (11%) had attempted to take their own lives (S3). However, among the 88 participants who attempted to take their lives, there were 79 (89.77%) participants who reported asking for medical help on the issue.

**Table 2:** Predictive model of suicide thoughts and attempts by demographics, psychological distress, loneliness, and social media addiction EL: emotional loneliness, SL: social loneliness S1: Have you ever had thoughts of taking your own life, even if you would not really do it? S2: During the past 12 months, have you had thoughts of taking your own life? S3: Have you ever attempted to take your own life?

	B	SE	Wald	df	Sig.	EXP(B)	95.0% CI for EXP(B)	
S1								
Age	-0.032	0.013	6.108	1	0.013	0.969	0.945	0.993
Kessler total score	0.06	0.009	42.569	1	<0.001	1.062	1.043	1.081
SL	0.369	0.085	18.873	1	<0.001	1.447	1.225	1.709
EL	0.293	0.088	11.066	1	<0.001	1.34	1.128	1.592
Constant	-2.348	0.418	31.628	1	<0.001	0.096		
S2								
Age	-0.068	0.021	10.808	1	0.001	0.935	0.898	0.973
Kessler total score	0.093	0.011	77.346	1	<0.001	1.098	1.075	1.121
SL	0.397	0.094	17.797	1	<0.001	1.488	1.237	1.789
Constant	-2.807	0.584	23.126	1	<0.001	0.06		
S3								
Age	-0.043	0.022	3.974	1	0.046	0.957	0.917	0.999
Social media addiction	-0.052	0.026	4.003	1	0.045	0.949	0.902	0.999
Kessler total score	0.04	0.013	9.794	1	0.002	1.041	1.015	1.068
EL	0.434	0.128	11.518	1	0.001	1.543	1.201	1.983
Constant	-1.991	0.746	7.118	1	0.008	0.137		

The final model of logistic regression analysis of S1 (“Have you ever had thoughts of taking your own life, even if you wouldn’t actually do it?) included psychological distress, EL, SL, and age. The model was significant (X2(4) = 150.889, p < 0.001) and explained 24.1% of the variation. In the model, age was inversely related to suicidal ideation, and SL was directly related to suicidal ideation. A 1-unit increase in the K10, EL, and SL each increased the odds of S1 by 1.06, 1.34, and 1.44, respectively. A one-year increase in age decreased the odds of EL and, therefore, of S1 by 0.969, as seen in Table 2.

For S2 (“During the past 12 months, have you had thoughts of taking your own life?”), the final logistic regression model of psychological distress, SL (direct relationships), and age (inverse relationship) and was statistically significant (X2(3) = 150.405, p < 0.001). The model explained 27.9% of thinking about suicide during the last 12 months. A 1‑unit increase in K10 and SL increased the odds of S2 by 1.098 and 1.488, respectively. A one-year increase in age decreased the odds of S2 by 0.935, as shown in Table 2.

For S3 (“Have you ever attempted to take your own life?”), the final logistic regression model included psychological distress, EL (direct relationships), age, and social media addiction (inverse relationships) and was statistically significant (X2(4) = 43.303, p < 0.001). The model explained 10.5% of suicide attempts. A 1-unit increase in K10 and EL increased the odds of suicide attempts by 1.041 and 1.543, respectively. A one‑year increase in age and social media addiction decreased the odds of a suicide attempt by 0.957 and 0.949 times, as shown in Table 2.

Suicidal questions were analyzed against the demographic data in Table 3, and the chi-square test and Fisher’s exact test were used for data analysis. Despite some of the demographic variables showing significant differences in a bivariable analysis, the multiple logistic regression found that none of the demographic variables (gender, nationality, specialty, qualifications, eating healthy food, or having chronic diseases) was significantly related to any of the three suicide questions.

**Table 3:** The relationship between demographic variables and suicidal questions using bivariable analysis *p-value <0.001 S1: Have you ever had thoughts of taking your own life, even if you would not really do it? S2: During the past 12 months, have you had thoughts of taking your own life? S3: Have you ever attempted to take your own life?

		S1		S2		S3	
Variable		Yes %	p-value	Yes %	p-value	Yes %	p-value
Gender	Male	25.23%	.002*	13.40%	0.003*	10.90%	0.943
	Female	35.49%		21.71%		11.06%	
Nationality	Saudi	32.04%	0.041*	18.68%	0.333	11.15%	0.761
	Non-Saudi	13.79%		10.34%		6.90%	
Region	Western	31.10%	0.09	18.90%	.0993	12.20%	0.239
	Central	33.82%		19.61%		9.80%	
	Southern	27.78%		12.50%		8.33%	
	Eastern	13.64%		4.55%		0.00%	
	Northern	60.00%		40.00%		20.00%	
Specialty	Dentistry	37.02%	0.452	18.23%	0.72	12.15%	0.766
	Public health	31.72%		20.15%		11.57%	
	Pharmacy	26.67%		13.33%		8.57%	
	Medicine	29.81%		19.88%		11.80%	
	Applied medical science	27.59%		17.24%		10.34%	
	Nursing	25.93%		14.81%		3.70%	
Qualification	student	35.10%	0.003*	21.41%	<0.001*	13.25%	0.043*
	Intern/resident	31.28%		20.00%		6.67%	
	specialist or consultant	20.39%		7.24%		9.87%	
Practice walking regularly	Yes	30.41%	0.651	20.95%	0.15	12.84%	0.203
	No	31.94%		16.87%		9.92%	
Eat healthy food	Yes	26.61%	0.248	18.35%	0.994	8.26%	0.325
	No	32.13%		18.38%		11.43%	
Having chronic disease	Yes	27.49%	0.047*	16.92%	0.371	11.18%	0.892
	No	34.12%		19.40%		10.87%	

## Discussion

The most widely used social media platforms, including Twitter, Instagram, and Facebook, have transformed how people communicate with each other, especially young people. There is evidence that those who spend a lot of time on social media exhibit more depression symptoms than those who spend only a small amount of time online [23].

Our study examined suicidal ideations and suicide attempts and their relationships with social media addiction, psychological distress, and loneliness among healthcare students and professionals in Saudi Arabia. Around one-third of participants reported having thought of taking their lives, approximately 2 out of 10 had that thought during the last year, and 1 in 10 healthcare students and professionals reported attempting to take their own life. The majority of those who attempted suicide reported previously asking for medical help. Psychological distress and loneliness were found to be significant factors that increased the odds of thinking about or attempting to commit suicide. However, older participants were less likely to think about or attempt suicide. Our study showed a higher prevalence of lifetime suicide thoughts than the general national population. The overall national rates were suicide attempts at 9.20%, plans to commit suicide at 3.10%, and lifetime suicide attempts at 2.70%, while in the Saudi population, they were 4.90%, 1.74%, and 2.03%, respectively [24]. Another study reported that 10.5 % of healthcare professionals overall had thoughts of suicide or self-harm, in comparison to our finding of 31.37% [25].

Literature indicated that the existing educational system might potentially exert an adverse impact on the mental well-being of students [26], especially medical students, who are reported to have high levels of depression, anxiety, and/or suicidal ideation [27]. Our findings were consistent with previous reports, such as from Aboalshamat et al., who reported that suicide ideation among medical students in the Eastern Province of Saudi Arabia was 42.2% over the course of the study period, while suicidal ideation within 12 months was 35.4% [28]. A 2021 study by Almoammar et al. was conducted in Abha (Southern Province) among dental students. The results indicated a suicide ideation rate of 43.5%, suicide ideation within 12 months of 30.7%, and a suicide attempt rate of 15.1% [13]. There is evidence that psychological distress and loneliness increase the chances of contemplating or attempting suicide. Madadin et al. conducted a 2021 study in the Eastern Province of Saudi Arabia and found that approximately one in three medical students in the study had suicidal ideations in the past 12 months, while around 40% had suicidal thoughts throughout their lives [12]. This issue can be accentuated by its association with confidentiality, time constraints, and the potential negative impact on healthcare worker careers [29].

A cross-sectional study by Mufti et al. was conducted in Madina, Saudi Arabia, in 2022 to identify the state of psychological well-being and its effect on suicidal behavior in 308 out of 1,397 medical students. Their results showed no correlation between psychological well-being and suicidal behavior. The study findings also showed that suicidal behavior was more prevalent among females [30]. Our findings also reflected lower rates of occurrence than the findings in another study that included healthcare students, which showed that 37.7% of their participants had experienced suicidal ideation throughout their lives. That is about double our results. Furthermore, their study revealed that 33.4% of participants (compared to our rate of 18.38%) had experienced suicidal ideation during the previous 12 months, and 23.2% of their participants (compared to our 11%) had attempted suicide [28].

Robles et al. reported in 2021 that, overall, suicidal ideation among healthcare workers was 13.8% [31]. This percentage was different according to the subcategories, which were 16.6% among undergraduate students, 15.9% among general practitioners and residents, 16% among specialists, 9.8% among nurses, and 17.4% among paramedics [31]. This is similar to our results in general; however, our results showed that there is no significant variation by qualification or specialty after including the other variables in the model. Thus, it might be important to conduct a multivariable analysis to identify significant variables.

The frequency of suicidal thoughts among healthcare workers may increase due to increasing burnout, posttraumatic stress disorder, and compassion fatigue expected with the next phase of the COVID-19 pandemic in the community, as well as the untreated mental health issues of healthcare workers who treat patients with COVID-19 [31]. Another study examined thoughts of suicide or self-harm among Australian healthcare workers in the midst of the COVID-19 pandemic, with results showing that 1 in 10 Australian healthcare workers expressed thoughts of suicide or self-harm, and certain groups were particularly at risk [25].

The results of a previous meta-analysis indicated a positive correlation between problematic Facebook use and psychological distress, which implies that social media addiction is connected to stress, anxiety, and depression [32]. In contrast, our study found that social media addiction might reduce the odds of attempting suicide. What is interesting about this finding is that the bivariate analysis showed that participants who attempted suicide had a higher social media addiction score. Surprisingly, however, the logistic multivariable analysis that included psychological distress, loneliness, and age indicated that the effect was protective. This finding broadly supports the work of Dhir et al., who reported in 2018 that social media addiction can induce exhaustion, resulting in depression and anxiety [33]. Social media addiction has been investigated based on mood management theory in order to determine whether social media use was beneficial to adolescents during the quarantine period in order to cope with feelings of anxiety and loneliness. In a survey of 2,165 Belgian adolescents, social media social relationships and humor were found to be a coping strategy for anxiety during the COVID-19 lockdown [34]. Our results cannot explain these findings. However, social media addiction might be a way to cope with loneliness among those participants, as found in a 2020 study by Boursier et al. [35].

Psychological distress and loneliness were found to be significant factors that can be risk factors for suicidal ideations and suicide attempts. This is supported by various previous reports that linked both factors to suicidal behaviors [8,9,13,14,28]. The findings of this study can provide theoretical evidence for implementing supportive psychological assistance to healthcare workers and students, setting guidelines for using social media to overcome the potential deterioration of social media addiction, psychological distress, and loneliness on suicidal ideations and suicide attempts among healthcare students and professionals in Saudi Arabia.

It is highly recommended that major action be taken to support healthcare students and professionals in dealing with psychological distress and loneliness because those factors can lead to serious consequences such as suicide. It should be highlighted that there are some efforts in Saudi Arabia to help healthcare professionals. For example, the Saudi Commission of Health Specialties offers the Daem program, which supports psychological residential programs [36]. Furthermore, the National Center for Mental Health Promotion provides free psychological consultations to Saudi residents [37]. Nevertheless, there is no clear evidence of the percentage of residents in the affected group using such services, especially with the highly persistent stigma of psychological problems among healthcare professionals, specifically [38,39].

Despite that we used a validated questionnaire in our study regarding suicidal thoughts, it might be beneficial to use other scales such as the Columbia Suicide Severity Rating Scale [40].

Our study has several strengths, including a diverse geographic distribution, a large sample size, and a coherent and representative sample. Despite the efforts in conducting this study, it is vulnerable to selection bias due to participant recruitment, which could jeopardize the external validity of the results. Finally, cohort and observational studies are required to further investigate the potentially complex interaction between social media use, psychological distress, loneliness, and suicide ideations and attempts because this was a cross-sectional study, in which causal inferences cannot be made.

## Conclusions

Healthcare practitioners are a vital, but vulnerable, segment of society. It is important to pay attention to the high rates of suicide ideation among them to determine the causes and take steps to prevent it. Determining healthy coping mechanisms is a priority that can help students get through the challenging times of specialty studies. The results of the current study showed a possible link between mental health issues and loneliness and suicidal behaviors in healthcare students and professionals. Research is needed to create efficient interventions to improve the psychological problems of the target population, and more focused efforts should be directed toward suicidal thoughts and suicide attempts. Multiple disciplines should be employed to analyze the relationship to the establishment of preventive measures. The positive relationship we discovered between social media use and suicide ideations and attempts, psychological distress, and loneliness has major implications for future studies and interventions given the rising rate of social media use and the significant morbidity and death associated with depressive disorders globally.
